# Consistent performance measurement of a system to detect masses in mammograms based on blind feature extraction

**DOI:** 10.1186/1475-925X-12-2

**Published:** 2013-01-10

**Authors:** Antonio García‐Manso, Carlos J García‐Orellana, Horacio González‐Velasco, Ramón Gallardo‐Caballero, Miguel Macías Macías

**Affiliations:** 1 Pattern Classification and Image Analysis Group, University of Extremadura, Avenida de Elvas s/n, Badajoz, Extremadura (SPAIN

**Keywords:** ICA, NN, SVM, CAD, Breast cancer, Mammography, DDSM

## Abstract

**Background:**

Breast cancer continues to be a leading cause of cancer deaths among women, especially in Western countries. In the last two decades, many methods have been proposed to achieve a robust mammography‐based computer aided detection (CAD) system. A CAD system should provide high performance over time and in different clinical situations. I.e., the system should be adaptable to different clinical situations and should provide consistent performance.

**Methods:**

We tested our system seeking a measure of the guarantee of its consistent performance. The method is based on blind feature extraction by independent component analysis (ICA) and classification by neural networks (NN) or SVM classifiers. The test mammograms were from the Digital Database for Screening Mammography (DDSM). This database was constructed collaboratively by four institutions over more than 10 years. We took advantage of this to train our system using the mammograms from each institution separately, and then testing it on the remaining mammograms. We performed another experiment to compare the results and thus obtain the measure sought. This experiment consists in to form the learning sets with all available prototypes regardless of the institution in which them were generated, obtaining in that way the overall results.

**Results:**

The smallest variation from comparing the results of the testing set in each experiment (performed by training the system using the mammograms from one institution and testing with the remaining) with those of the overall result, considering the success rate for an intermediate decision maker threshold, was roughly 5%, and the largest variation was roughly 17%. But, if we considere the area under ROC curve, the smallest variation was close to 4%, and the largest variation was about a 6%.

**Conclusions:**

Considering the heterogeneity in the datasets used to train and test our system in each case, we think that the variation of performance obtained when the results are compared with the overall results is acceptable in both cases, for NN and SVM classifiers. The present method is therefore very general in that it is able to adapt to different clinical situations and provide consistent performance.

## Background

As was observed in [1], to detect and diagnose lesions (mainly masses and microcalcifications) in mammograms, a CAD system needs to satisfy various quality criteria: high sensitivity to detect the greatest possible number of cancers; high specificity to reduce the frequency of false positives per image; acceptable call rate; early detection to increase the patient’s chances of survival; fast processing time; and robustness. This last is in the sense that the system should be adaptable to different clinical situations and should provide consistent performance. We have designed and implemented a system based on blind feature extraction by Independent Component Analysis (ICA) and Neural Network (NN) and Support Vector Machine (SVM) classifiers to detect and classify masses in the mammograms of the Digital Database for Screening Mammography (DDSM) [2].

Many methods have been proposed in the last two decades to achieve robustness in mammography‐based computer aided detection (CAD) systems. Although there are various types of mammographic abnormalities, they can primarily be categorized as either masses or microcalcifications [3]. Many of the proposals in the literature focus on the detection and segmentation of masses on mammograms. Some examples are reviewed in [4]. But it is usually difficult to compare the results of different studies addressing both the detection and diagnosis of masses, the main problem being either the use of small‐size proprietary databases or, if they use a public database, the use of selected, unspecified cases.

Horsch [5], in an analysis of recent mammography CAD studies, concludes that, in view of the observed variability in the datasets used, currently the only mammography database that is both public and sufficiently large to allow a meaningful and reproducible evaluation of a CAD system is the DDSM. The DDSM contains mammograms obtained from examinations between October 1988 and February 1999 at four different clinics: Massachusetts General Hospital (MGH) in Boston; Wake Forest University School of Medicine (WFU) in North Carolina; Sacred Heart Hospital in Pensacola (SH), Florida; and Washington University School of Medicine in St. Louis Medical Center (WU).

These mammograms were digitized using four different makes of scanner: DBA M2100 ImageClear with a resolution of 42*μm*; Howtek with a resolution of 43.5*μm*; Lumisys 200 laser with a resolution of 50*μm*; and Howtek MultiRAD 850 with a resolution of 43.5*μm*. This gives an idea of the underlying heterogeneity of the dataset we used. To normalize the dataset and thus avoid this heterogeneity, we used the calibration curves which are available for each scanner to obtain the mammographic images in optical densities. In that way, at least in theory, the entire dataset would be normalized to the same conditions. But the number of prototypes for each class digitized with each scanner may differ widely from one scanner to another. And the way in which ground truth for the mammograms was indicated could also be very different [5]. One reason clearly was the long period of time (more than 10 years) during which DDSM was constructed. Another was that the radiologists used different styles when indicating the lesions on the mammograms, firstly, because obviously many radiologists were involved, and secondly because the styles of the reports corresponded to four different institutions.

We here propose a system to discriminate masses from normal tissue as a two‐class pattern recognition problem (mass or normal tissue), but without the use of any modeling. Instead, we use ICA‐based blind feature extraction for its ability to obtain basis functions well adapted to the problem, especially to natural images [6,7]. In particular, we obtain basis functions (i.e., basis images) in which to decompose the original image (original patch), and then use the coefficients of this decomposition to form the input vectors to the classifiers.

The rest of our paper is organized as follows. Section “*Methods*” presents the methodological approach taken, including a description of the general concepts of feature extraction and classifiers, and of the dataset used in our trials. Section “*Outline of the process*” describes the proposed system, and Section “*Results*” presents the results. Finally, Section “*Conclusions*” gives the main conclusions of the work.

## Methods

As stated above, the aim of this work is to assess the robustness of our system, focusing here our attention in the classifying of regions of interest. Our system consist in a two‐class pattern recognition problem (mass or normal tissue) based on feature extraction and classification of regions of interest (previously extracted from the DDSM database, in our experiments). As explained above, a conversion of the image to optical density is made first, as a preprocessing stage. Later, the regions of interest are resized to the appropriate size for the feature extractor, which is based in blind feature extraction using independent component analysis. Afterwards, the classification stage is carried out using neural network classifiers or support vector machine classifiers.

In this section, we shall provide a brief description of the mammogram database utilized, of the procedure implemented to build a set of mass and normal tissue prototypes (regions of interest, ROIs), and of the main characteristics of the selected image feature extractor. Finally, we shall briefly describe the classifiers used.

### Data and prototype creation

The DDSM [2] is a resource made available to the mammographic image analysis research community. It contains a total of 2620 cases, each of which provides four screening views – mediolateral oblique (MLO) and craniocaudal (CC) projections of the left and right breasts. The database therefore has a total of 10 480 images. The cases are categorized into four major groups: *normal, cancer, benign*, and *benign without callback*. The experienced radiologists that participated in the elaboration of the DDSM provided BIRADS parameters (density, assessment and subtlety), the BIRADS abnormality description, and the proven pathology, for all the cases in the database. For each abnormality identified, the radiologists drew free‐form digital curves defining ground truth regions. We use these regions to define square regions‐of‐interest (ROIs) for use as mass prototypes. Each DDSM case includes additional information – patient age, date of study, and the make or brand name of the digitizer.

**Figure 1:**
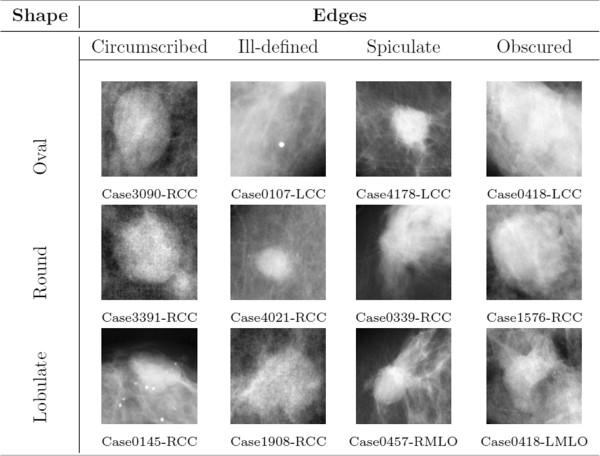
**Main types of masses: Examples of masses for each combination of shape and edges.** Each ROI image has been re‐sized to a common size of 128 × 128 *pixels*, to show them here as example. The case name and view is given below each ROI.

The DDSM database contains 2582 mass prototypes including both benign and malignant masses. Some are located on the border of the mammogram, and could not be used (see the following paragraph, dedicated to ROIs). Consequently, only 2324 prototypes were considered, namely, those which could be taken centred in a square without stretching. Some mass prototype examples are shown in Figure 1.

#### Regions of Interest

**Figure 2:**
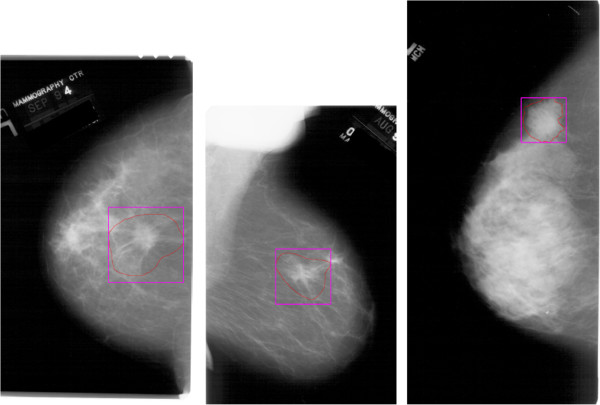
**Examples of ground truth over mammograms.** This figure shows the ground truth regions included in DDSM database (solid red curve, manually coded by a radiologist) for three different examples, and the area to be used as ROI (purple square box), in each case.

Ground truth regions are defined in the database by a chain code which generates a free‐hand closed curve. We use the chain code to determine the smallest square region of the mammogram that includes the manually defined region. Therefore, if the mass is located near one edge of the mammogram, this procedure may not be able to obtain a square region from the image, and the mass is then discarded from further consideration as a valid prototype. Figure 2 shows the ground truth regions coded by radiologists (solid red curve) in three different examples, and the area to be used as ROI (purple square box), in each case.

The DDSM mammograms were digitized with four different scanners of known optical density calibration and spatial resolution [2]. Three of the scanners provide a linear optical response, and the fourth a logarithmic response. To eliminate the dependence of the digitized mammograms on the origin, all the ROIs obtained were converted to optical densities using the referenced calibration parameters.

The regions generated are of different sizes. Therefore, since the chosen image feature extractor needs to operate on regions of the same size, the selected regions had to be reduced to a common size. Such reduction of the ROIs to a common size has been demonstrated to preserve the mass malignancy information [8]‐[10]. To determine the optimum region size, we re‐sized each ROI to two sizes: 32×32 and 64×64 pixels. We also tried other sizes such as 128 × 128 pixels, but the performance obtained with this size was not better than that obtained with the two smaller sizes, whereas the computation time was much greater. The re‐sizing was carried out using the bilinear interpolation algorithm provided by the OpenCV library [11].

### Independent Component Analysis

Independent Component Analysis could be considered to be the next step beyond Principal Component Analysis (PCA) [12]. Its original motivation was to solve problems known as *blind source separation* (BSS). In particular, suppose that one has *n* signals. The objective is to expand the signals registered by the sensors (**x**_*i*_) as a linear combination of *n* sources (**s**_*j*_), in principle unknown [13].

(1) $$x_{i}=\overset{n}{\underset{j=1}{\sum }}a_{\text{ij}}s_{j}$$

The goal of ICA is to estimate the mixture matrix **A** = (*a*_*ij*_), together with the sources **s**_*j*_. The ICA model assumes that the observed signals are a linear transformation of some hidden sources: **x = A · s**. In general, the mixture matrix **A** is invertible, so that one has:

(2) $$x=A\cdot s\Rightarrow s=W\cdot x\;\text{with}\;W=A^{-1}$$

It is important to remark that: 

• The key assumption of ICA estimation is that the hidden sources (**s**) are non‐Gaussian and statistically independent.

• One cannot determine the variances (energies) of the independent components. Therefore, the magnitudes of the **s**_*i*_ can be freely normalized. And neither can one determine the order of the independent components.

This technique can be used for feature extraction since the components of **x** can be regarded as the characteristics representing the objects (patterns) [13]. We use the FastICA algorithm [14], proceeding as follows: 

• We start with N samples (N patches (vectors) of dimension *p*) forming the *N* × *p* patches matrix.

• First, we centre the data by subtracting their means, i.e., from each element is subtracted the mean of its column.

• To reduce the size of the input space we apply PCA, ordering the array of eigenvectors by its eigenvalues from highest to lowest and discarding those with lower eigenvalues, which will be those making a smaller contribution to the variance. Taking *q* (*q* < *p*) first components, we obtain the matrix **K**_*PCA*_ of dimension (*q* × *p*).

• Now, taking as input this matrix and applying the ICA algorithm, we obtain the ICA transformation matrix, **W**, of dimension (*q* × *q*).

• Finally, considering a new matrix (**W**_*T*_), a product of the previous two ($W_{T}=K_{\text{PCA}}^{T}\cdot W$ (*p* × *q*)), in which each row is a vector of the new base, we can extract *q* characteristics of each original input simply by multiplying the matrix **W**_*T*_ by each of these inputs:

(3) $$s=i\cdot W_{T}$$

**Figure 3:**
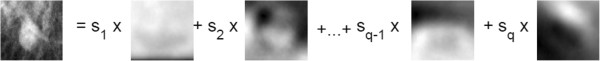
Decomposition of the image using an ICA base: The figure shows how an image can be decomposed using the inverse of an ICA base (image basis) and the eigenimage coefficients obtained by applying that base.

As is also the case with many other transformations (wavelets, Gabor filters,... [15]), following this process we can express the image (or image patch) as a linear superposition of some basis functions (basis images in our case) *a*_*i*_(*x*, *y*):

(4) $$I(x,y)=\overset{q}{\underset{j=1}{\sum }}a_{j}(x,y)s_{j}$$

where the **s**_*j*_ are image‐dependent coefficients. This expression is similar to the ICA model, and the idea is illustrated with specific images in Figure 3. In particular, the figure shows how an image can be decomposed using the inverse of an ICA base (image basis) and the eigenimage coefficients obtained by applying that base. In this way, by estimating an image basis using **ICA**, one can obtain a base adapted to the data at hand.

### Classification algorithm

In our system, the classification algorithm has the task of learning from data. An excessively complex model will usually lead to poorly generalizable results. It is advisable to use at least two independent sets of patterns in the learning process: one for training and another for testing. In the present work, we use three independent sets of patterns: one for training, one to avoid overtraining (validation set), and another for testing [16]. For the classification, we use NN and SVM classifiers [17].

#### Neural Networks

We implement the classical feed‐forward multilayer perceptron (BP) with a single hidden layer, and a variant of the Back‐Propagation algorithm termed Resilient Back‐Propagation (Rprop) [18] to adjust the weights. This last is a local adaptive learning scheme performing supervised batch‐learning in a multilayer perceptron which converges faster than the standard BP algorithm. The basic principle of Rprop is to eliminate the negative effect of the size of the partial derivative on the update process. As a consequence, only the sign of the derivative is considered in indicating the direction of the weight update [18]. The function library of the Stuttgart Neural Network Simulator environment [19] is used to generate and train the NN classifiers. To avoid local minima during the training process, each setting was repeated four times, changing the initial weights in the net at random. Furthermore, the number of neurons in the hidden layer was allowed to vary between 50 and 650 in steps of 50.

#### Support Vector Machines

The goal of using an SVM is to find a model (based on the training prototypes) which is able to predict the class membership of the test subset’s prototypes based on the value of their characteristics. Given a labeled training set of the form (**x**_*i*_, **y**_*i*_), *i* = 1, …, *l* where $x_{i}\in R^{n}$ and **y** ∈ {1, -1}^*l*^, the SVM algorithm involves solving the following optimization problem:

(5) $$\begin{array}{lc} \underset{w\in R^{d},b,\xi_{i}\in R^{+}}{\text{min}} & {∥w∥}^{2}+C\overset{l}{\underset{i=1}{\sum }}\xi_{i}\\ \text{subject to} & y_{i}\left(w^{T}\phi \left(x_{i}\right)+b\right)\geq 1-\xi_{i},\\ & \xi_{i}\geq 0 \end{array}$$

In this algorithm, the training vectors **x**_*i*_ are projected onto a higher‐dimensional space than the original. The final dimension of this space depends on the complexity of the input space. Then the SVM finds a linear separation in terms of a hyperplane with a maximal (and hence optimal) margin of separation between classes in this higher dimensional space. In the model, *C* (*C* > 0) is a regularization or penalty parameter to control the error, *d* is the final dimension of the projection space, **W** is the normal to the hyperplane (also known as the weights vector), and *b* is the bias. The parameter *ξ* is introduced to allows the algorithm a degree of flexibility in fitting the data, and *K*(**x**_*i*_, **x**_*j*_) ≡ *ϕ*(**x**_*i*_)^*T*^*ϕ*(**x**_*j*_) is a kernel function to project the input data onto to a higher dimensional space. We used the LibSVM [20] library with a radial basis function (RBF: *K*(*x*_*i*_, *x*_*j*_) = exp(-*γ* ∥*x*_*i*_ - *x*_*j*_∥^2^), *γ* > 0) as kernel function. To find the optimal configuration of the parameters in the algorithm, *γ*, was allowed to vary between -5 and 20 in steps of 0.5, and the penalty parameter *C* between ‐5 and 10 also in steps of 0.5.

### Outline of the process

In this section, we provide an overview of the structure of our system, describing the main steps required to configure the system to discriminate prototypes of masses from prototypes of normal tissue.

#### System description

The aim of this study is to evaluate the performance that our system can provide for detection of masses based on blind feature extraction using ICA and, using as classifiers, neural networks and SVM.

**Figure 4:**
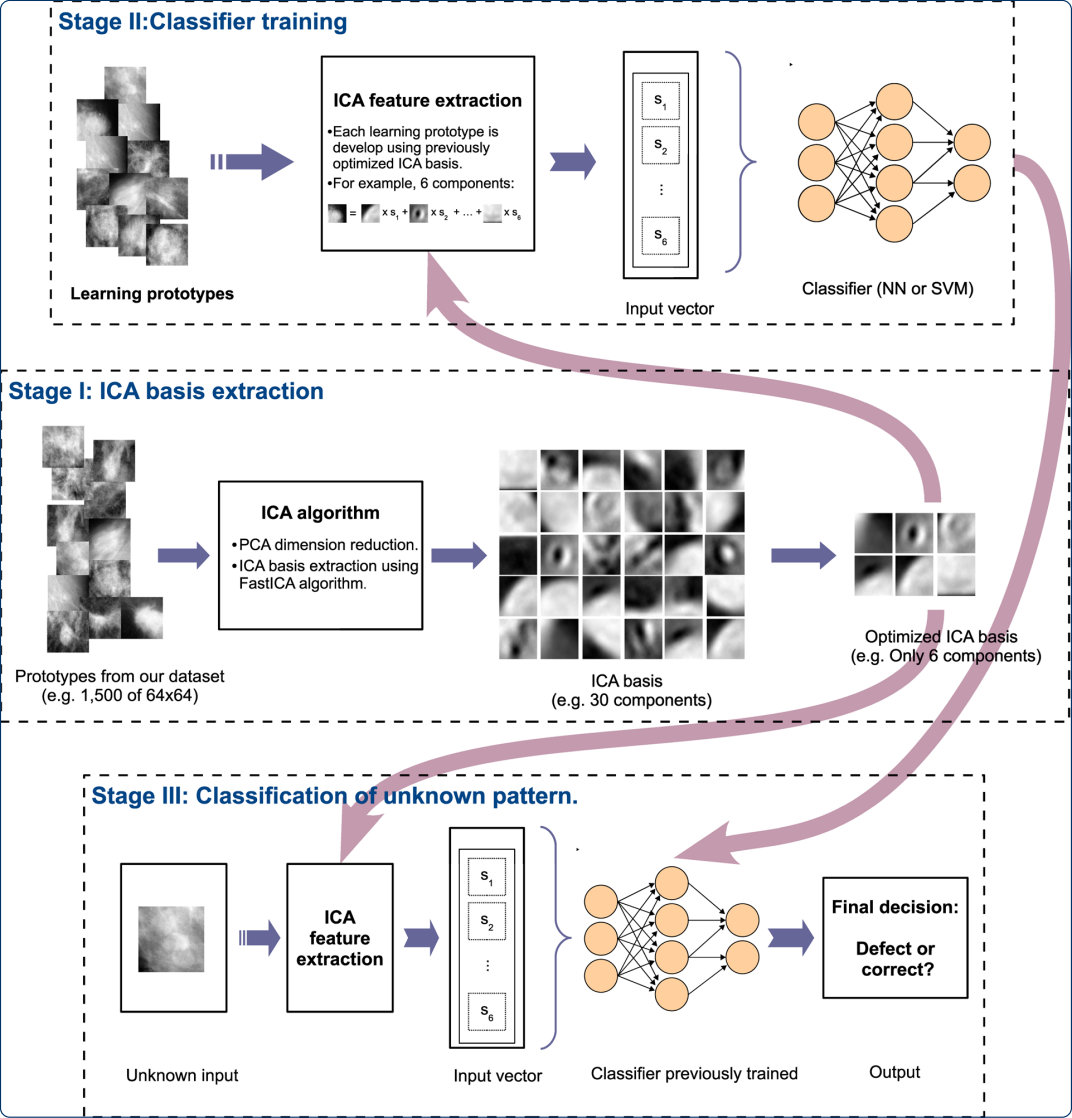
Overview of the system: The figure shows the main scheme that summarizes in a graphical form all phases of this work.

The main scheme that summarizes in a more graphical form all phases of this work is represented in Figure 4. In the *first stage*, the prototypes of masses are obtained as was explained in Section “*Regions of Interest*”, and those of normal tissue were selected at random from the normal mammograms. These normal tissue prototypes were initially captured with sizes ranging randomly from the smallest to the largest sizes found in the DDSM for masses. Then the FastICA algorithm [14,21] is applied as described in Section “*Independent Component Analysis*” to obtain the ICA base (the ICA‐based feature extractor), with the *log cosh* function being used to approximate the neg‐entropy. These basis are generated with different configurations, different numbers of components, and using prototypes of different sizes. The *second stage* uses this generated basis to obtain the training sets and to train and test the classifiers. Finally, in the *third stage*, the test subset, which contains input vectors not used in the optimization of the classifiers, is used to provide performance results of our system.

#### System optimization

To determine the optimal configuration of the system, various ICA bases were generated to extract different numbers of features (from 10 to 65 in steps of 5) from the original patches, and operating on patches of the different sizes noted above (32 × 32 and 64 × 64 pixels).

**Figure 5:**
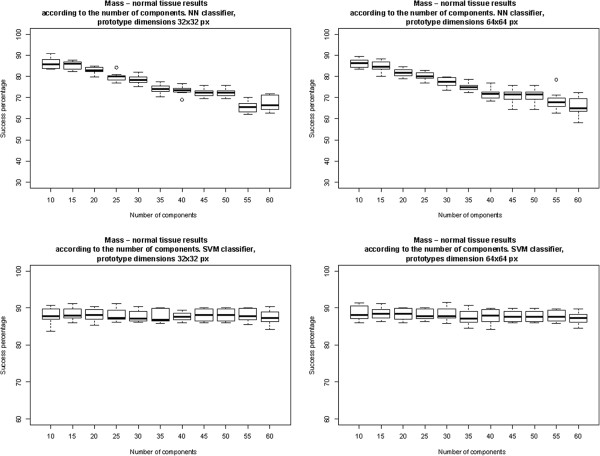
**System optimization: Choosing the best configuration for the feature extractor.** The top row shows the results when using an NN classifier, and the bottom row, the results for an SVM classifier. In both cases, prototypes of 32 × 32 are in the first column, and of 64 × 64 in the second column.

The training process was performed two times – first training the NN classifiers, and then the SVM classifiers. The results thus obtained on the test subsets in a 10‐fold cross validation scheme are shown in Figure 5. This allowed us to find the optimal configuration of the feature extractor.

The study was done with a total of 5052 prototypes: 1197 of malignant masses, 1133 of benign masses, and 2722 of normal tissue.

We found that the optimal ICA‐based feature extractor configuration for an NN classifier was a feature extractor that operated on prototypes of 64 × 64 pixels, extracting 10 components (average success rate 86.33%), and for an SVM classifier was a feature extractor that also operated on prototypes of 64 × 64 pixels, extracting 15 components (average success rate 88.41%). The results to be presented in the following section were obtained using these optimal configurations.

## Results

In this section, we provide an description of the experiments made to measure the robustness of our system and the results obtained.

### Experiments

The principal objective of the present work was to evaluate the robustness of our system to discriminate masses from normal tissue. For this, we used the prototypes of masses and normal tissue described above. All these prototypes coming from four different clinics and, in each clinic was used a specific kind of scanner (as stated above). Therefore, these prototypes were divided according to the scanner used for scanning. Our experiment consists first in to train and test the system using all available prototypes divided at random into the learning and test sets, in that way, we obtain the overall results. And, then, the system is trained and optimized each time using only the prototypes coming from one clinic (digitized with one specific scanner) and, the system performance is tested with the prototypes coming from the other clinics. In that way, we can assess the robustness of the system comparing the results obtained with each configuration. The distribution of the prototypes in each configuration is given in Table 1.

**Table 1:** Distribution of prototypes for the different scanners

**Distribution of prototypes for the different scanners**			
**Scanner**	**Pathology**	**Learning**	**Test**
**HOWTEK 960** (MGH)	Malignant	345	851
43.5*μm* / *pixel*	Benign	485	648
linear calibration	Normal	312	2410
**HOWTEK MultiRAD 850** (WU)	Malignant	107	1089
43.5*μm* / *pixel*	Benign	154	979
linear calibration	Normal	417	2305
**DBA M2100** (MGH)	Malignant	105	1091
42*μm* / *pixel*	Benign	0	1133
logarithmic calibration	Normal	1668	1054
**LUMISYS 200 laser** (WFU & SH)	Malignant	639	557
50*μm* / *pixel*	Benign	494	639
linear calibration	Normal	325	2397
	Malignant	1077	119
Overall	Benign	1028	105
	Normal	2441	281

Distribution of prototypes in the learning and test sets for the different scanners used, and without considering the scanner (overall results). In parentheses next to the scanner’s name is the identification of the institutions in which that make of scanner was used.

As one observes in that table, the number of prototypes in the learning and test sets is quite dependent on the scanner, the most heterogeneous distribution being for the DBA M2100 scanners. With this make of scanner, no prototypes of benign masses were found, and there was far fewer prototypes of malignant masses than of normal tissue in the learning set. As will be seen below, this is a major handicap in training the classifiers.

Table 1 also shows that the DBA scanner provided more than half of the total of normal tissue prototypes (1668 DBA scanner normal prototypes as against 2440 total normal prototypes). This is because there are 12 volumes of normal mammograms in the DDSM, each with different numbers of cases, and with four mammograms per case. Of these 12 volumes, 6 were digitized with a DBA scanner, among them those with the largest number of cases. Therefore, if we had selected the normal tissue prototypes at random from the normal mammograms, then more than half would have been from a DBA scanner. However, there were only two volumes of *“cancer”* from a DBA scanner, and none of *“benign”*. The numbers of prototypes are more evenly distributed among the rest of the scanners.

### Results

**Figure 6:**
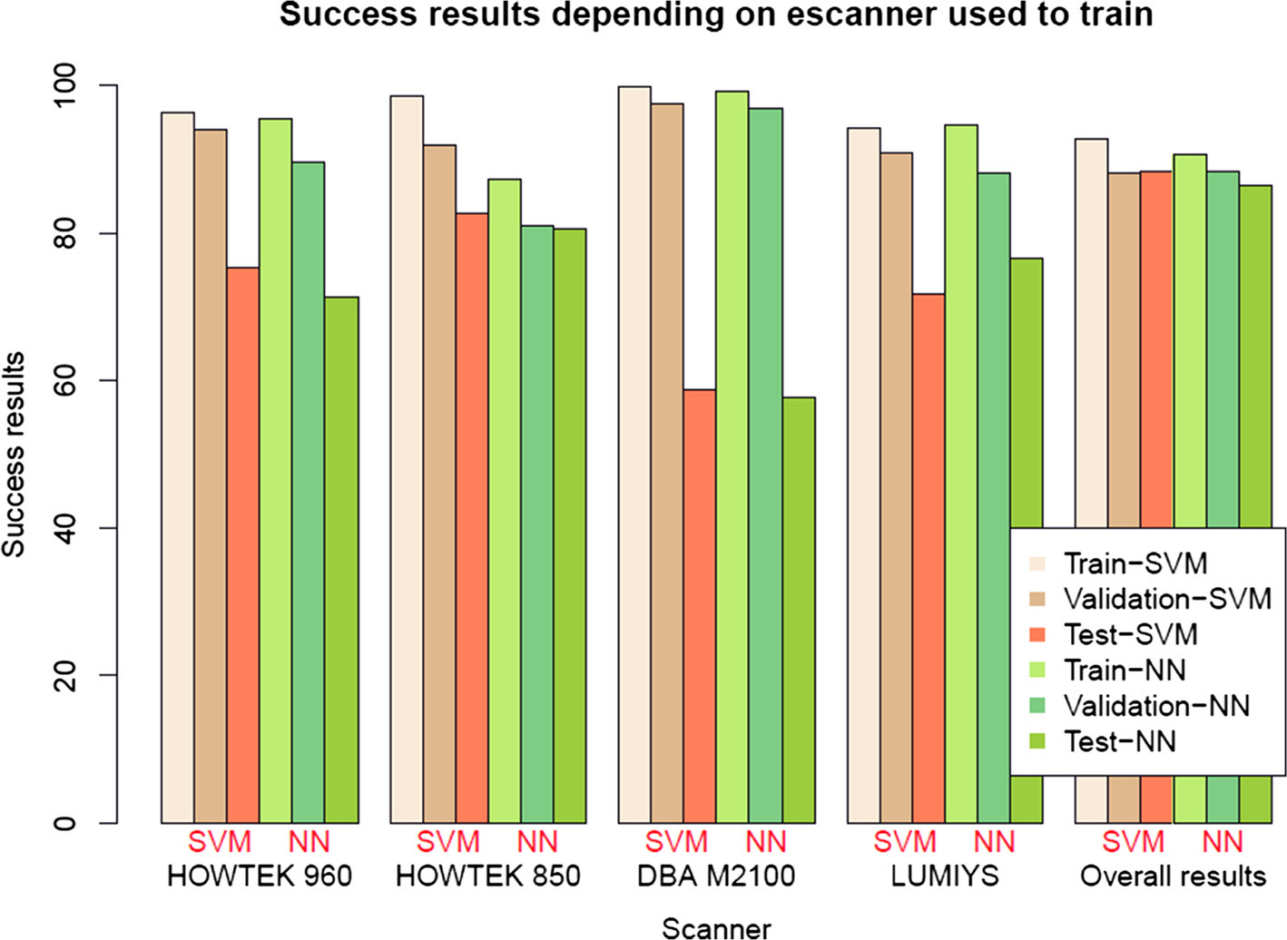
**Dependence of the final success rates on the mammograms selected to train the system: The dependence of the final success rates on the mammograms selected to train the system.** For each of the four makes of scanner named below the sets of histograms, the learning set consisted only of mammograms digitized by that scanner.

**Figure 7:**
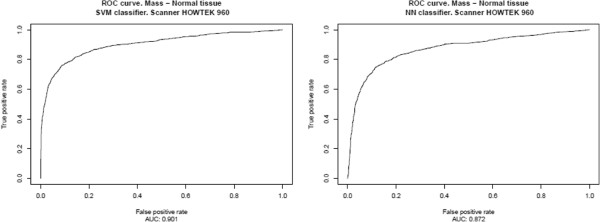
HOWTEK 960: Results obtained over the test subset (formed for all prototypes minus those from HOWTEK 960 scanner), considering only the prototypes from HOWTEK 960 scanner to form the learning set.

**Figure 8:**
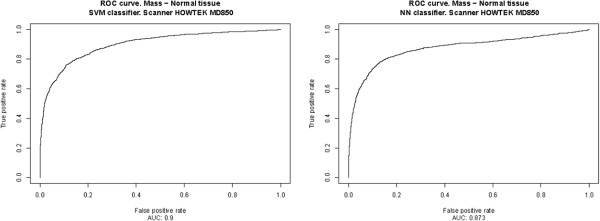
HOWTEK 850: Results obtained over the test subset (formed for all prototypes minus those from HOWTEK MD850 scanner), considering only the prototypes from HOWTEK MD850 scanner to form the learning set.

**Figure 9:**
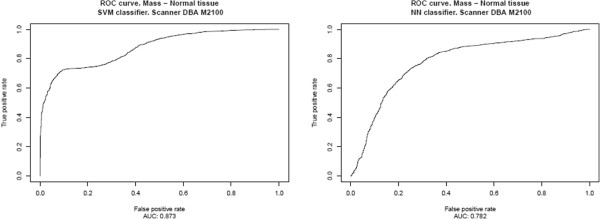
DBA M2100: Results obtained over the test subset (formed for all prototypes minus those from DBA M2100 scanner), considering only the prototypes from DBA M2100 scanner to form the learning set.

**Figure 10:**
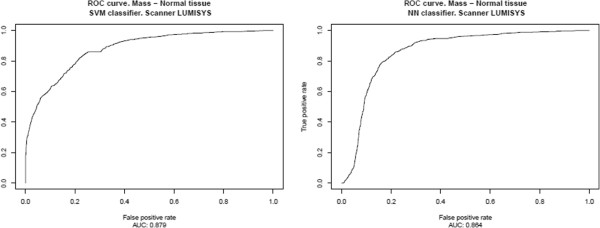
LUMYSIS: Results obtained over the test subset (formed for all prototypes minus those from LUMISYS scanner), considering only the prototypes from LUMISYS scanner to form the learning set.

**Figure 11:**
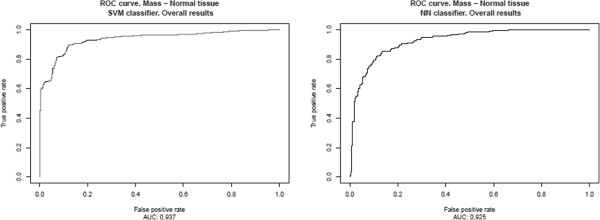
Overall results: Results obtained over the test subset considering all available prototypes, regardless of their origin.

The results, presented in Table 2 and Figure 6, correspond to the ICA‐based feature extractors described in the previous section, Section “*System optimization*”, for each classifier, using an intermediate decision maker threshold in both cases. The results for all possible decision maker thresholds are shown in Table 3 and in Figures 7, 8, 9, 10, 11, using ROC analysis [22] as is recommended in [23].

**Table 2:** Success results

**Success results**						
	**SVM classifier**			**NN classifier**		
**Scanner**	**Learning (%)**		**Test(%)**	**Learning(%)**		**Test(%)**
	Train(80%)	Val(20%)		Train(80%)	Val(20%)	
HOWTEK 960	96.38	93.89	75.29	95.40	89.52	71.22
HOWTEK MultiRAD 850	98.71	91.91	82.71	87.27	80.88	80.56
DBA M2100	99.80	97.46	58.69	99.29	96.90	57.75
LUMISYS	94.17	90.75	71.72	94.68	88.01	76.62
Overall results	92.31	88.22	86.93	90.88	86.14	84.95

The final success rates. The learning set is divided into two: the training subset (Train) and the validation subset (Val), corresponding to 80% and 20% of the prototypes in the learning set, respectively. For each of the four scanners named in the leftmost column, the learning set consisted only of mammograms digitized by that make of scanner.

**Table 3:** Area under ROC curve

**Mass ‐ normal tissue**		
**Depending on the origin of mammograms**		
	**AUC**	
**Scanner**	**SVM classifier**	**NN classifier**
HOWTEK 960	0.901	0.872
HOWTEK MultiRAD 850	0.900	0.873
DBA M2100	0.873	0.782
LUMISYS	0.879	0.864
Overall results	0.937	0.925

This table shows the results obtained over the different test subsets (considering all the prototypes to form the learning set, or considering only the prototypes from one scanner to form the learning set), as area under the ROC curve (AUC)

One observes that the most appropriate distribution of prototypes is that corresponding to the overall results. This is because for this choice there are far more prototypes in the learning set than in the test set (Table 1), and the classifier trained with this distribution can *“learn”* prototypes from all the scanners (from all four clinics), while, those trained with the other distributions can only learn prototypes from one scanner (from one clinic). Nevertheless, as was noted above, in theory, the scanner used for scanning should not affect the final results because the prototype images are transformed to optical densities using the scanner calibration parameters provided by the DDSM’s authors, but yes the different styles used for indicating the lesions on the mammograms and, the number of prototypes in the learning set in each case.

The performance obtained in each case can be evaluated by taking as referent that with the overall test subset, because in this case the learning set consisted of prototypes of all four makes of scanner, and the classifiers can *learn* prototypes corresponding to all of them. With this choice of referent and considering the results shown in Table 2, for an SVM classifier the differences in performance were: 13.4% for Howtek 960, 4.8% for Howtek MultiRAD 850, 32.5% for DBA, and 17.5% for Lumisys. And for an NN classifier they were: 16.2% for Howtek 960, 5.2% for Howtek MultiRAD 850, 32.02% for DBA, and 9.8% for Lumisys. Given the aforementioned recognized problems with the DBA scanner, the results for this scanner have to be regarded as inconclusive.

On the other hand, in Table 2 and Figure 6, one observes that when there are relatively few prototypes in the learning set (the HOWTEK scanners) the performance with SVM classifiers is slightly better than with NN classifiers. This seems to be in agreement with the results of [24] in which a comparison was made of the performance and robustness of different types of classifiers in different settings. In contrast, when the numbers of the different types of prototype are relatively large (the LUMISYS scanners), although the performance with NN classifiers may at first sight seem to be slightly better than with SVM classifiers, this is not true, since the performance for the case of the overall results (case with the largest number of prototypes in the learning set) is somewhat better with SVM classifiers. And, we also can observe this made analyzing the results obtained by mean of the ROC analysis. From these results, shown in Table 3 and in Figures 7, 8, 9, 10, 11, one can see that the SVM classifiers perform better than NN classifiers in all cases, particularly when only the cases from MGH (DBA scanner) were used to form the learning set, which yielded a difference of about 10% in the AUC.

## Conclusions

The robustness of our system has been studied using a very heterogeneous dataset. The data (mammograms and reports) originated from four clinics at which the mammograms were digitized using, at first, four different makes of scanner, although the DBA scanner, used at MGH, was retired due to continuing performance difficulties [25]. Considering this heterogeneity in the datasets used to train and test the system in each case, we think that the variation of performance obtained when the results are compared with the overall results (best convenient distribution of the prototypes) is relatively low in both cases, for NN and SVM classifiers. The smallest variation in performance was found for the Howtek MultiRAD 850 scanner with both classifiers, suggesting that the entire dataset was well represented by the images digitized with this scanner.

On the other hand, from the results obtained by mean ROC analysis we can say that our system to discriminate between prototypes of masses and normal tissue yields better with SVM classifiers than with NN classifiers.

## Abbreviations

NN: Neural Network; SVM: Support Vector Machine; ICA: Independent Component Analysis; CAD: Computer Aided Detection; DDSM: Digital Database for Screening Mammography; MGH: Massachusetts General Hospital; WFU: Wake Forest University School of Medicine; SH: Sacred Heart Hospital.

## Competing interests

The authors declare that they have no competing interests.

## Authors’ contributions

AGM developed the pre‐processing system (selection and acquisition of ROIs, and obtaining the ICA bases), integrated the global system, conducted the experiments, obtaining and analyzing the results, and drafted the manuscript. CJGO developed the neural network classifier training algorithm, helped to adapt and adjust the hardware for the simulations (two Beowulf clusters with 45 and 48 nodes, respectively), together with the adaptation of the software to be run on the clusters. RGC developed the database associated with the experiments. HGV was responsible for the assembly and tuning of the clusters. MMM developed the support vector machine classifier training algorithm. All authors read and approved the final manuscript.
